# Characterization and antimicrobial activity of a novel lytic phage vB_SmaS_QH16 against *Stenotrophomonas maltophilia*: *in vitro*, *in vivo*, and biofilm studies

**DOI:** 10.3389/fcimb.2025.1610857

**Published:** 2025-07-10

**Authors:** Peng Cheng, Zian Li, Lanmin Liu, Ruizhe Li, Jianwu Zhou, Xiaoqin Luo, Xiaoming Mu, Jingwei Sun, Jideng Ma, Xiangren A

**Affiliations:** ^1^ College of Clinical Medicine Qinghai University, Xining, China; ^2^ Department of Clinical Laboratory, Qinghai Provincial People’s Hospital, Xining, China

**Keywords:** *Stenotrophomonas maltophilia*, phage, genomic analysis, biofilm, phage therapy

## Abstract

**Background:**

*Stenotrophomonas maltophilia*, an important opportunistic pathogen resistant to multiple antibiotics, necessitates alternative therapies. Phages, with their high specificity and bacteriolytic ability, are emerging as promising antibiotic alternatives. This study aimed to isolate and characterize a novel lytic phage targeting *S. maltophilia* and to evaluate its antibacterial potential.

**Methods:**

A novel lytic phage, vB_SmaS_QH16, was isolated from hospital sewage using *S. maltophilia* no.981 as the host. Phage morphology was analyzed using transmission electron microscopy (TEM), and genome sequencing and annotation were performed. Host range, efficiency of lysis (EOP), optimal multiplicity of infection (MOI), one-step growth curves, and physicochemical stability were also determined. Biofilm inhibition and eradication were assessed using crystal violet staining, MTT assays, and acridine orange fluorescence microscopy. Using *Galleria mellonella* and mouse infection models, the *in vivo* anti-infective effects of phages were evaluated.

**Results:**

Phage vB_SmaS_QH16, a member of the class Caudoviricetes, has a 43,500 bp genome with 64 open reading frames (ORFs) and no virulence, antibiotic resistance, or lysogeny-related genes. It exhibits a broad host range, lysing 47.95% (35/73) of tested *S. maltophilia* strains. The optimal MOI was 0.01, with an average burst size of 37.69 PFU/cell. The phage is stable at 4–50 °C and pH 3.0–11.0 but is highly sensitive to UV light. It effectively inhibits biofilm formation and eradicates mature biofilms in a concentration-dependent manner. *In vitro*, the phage significantly suppresses bacterial growth, though resistant mutants emerge over time. *In vivo*, vB_SmaS_QH16 increases the survival rates of larvae and mice, with a higher MOI offering better protection.

**Conclusions:**

Phage vB_SmaS_QH16 shows therapeutic potential against *S. maltophilia* infections, characterized by a broad host range, efficient lytic capability, and biofilm-disrupting activity. Its stability and safety further support its clinical application potential. Future research should explore its biofilm disruption mechanisms and monitor resistance development. Additionally, since its efficacy has been validated in mammalian models, further studies can focus on advancing its clinical translation.

## 1 Introduction

Antimicrobial resistance has emerged as a critical global public health challenge in the 21st century, posing major threats to human health and survival (Howard et al., 2014; Prestinaci et al., 2015). *Stenotrophomonas maltophilia*, a Gram-negative, non-fermentative bacterium ubiquitously distributed in natural and healthcare environments, has recently gained prominence as an important opportunistic pathogen due to its intrinsic and acquired multidrug resistance mechanisms (Majumdar et al., 2022). Although this species generally exhibits lower pathogenicity than other nosocomial microorganisms, its biofilm-forming capacity and virulence factors enable colonization and infection in immunocompromised hosts, particularly those with underlying pulmonary comorbidities or hematologic malignancies, most commonly in intensive care settings. Epidemiological studies report attributable mortality rates of up to 38% for *S. maltophilia* infections, increasing to over 50% in cancer patient cohorts (El Chakhtoura et al., 2018). Predisposed populations include chemotherapy recipients and chronic respiratory disease patients, with lower respiratory tract infections predominating clinical manifestations—notably chronic obstructive pulmonary disease exacerbations, hospital-acquired pneumonia, and ventilator-associated pneumonia (Brooke, 2012).

The therapeutic arsenal against *S. maltophilia* remains limited. Sulfamethoxazole/Trimethoprim (SXT) constitutes the first-line therapy, followed by levofloxacin (LEV) as a secondary option (Nicodemo and Paez, 2007). The bacterium’s inherent resistance mechanisms, mediated through acquired resistance genes/plasmids and multidrug efflux pumps, have precipitated the rapid development of resistance to previously effective agents (Hu et al., 2011). This alarming trajectory underscores the imperative for novel antimicrobial strategies to safeguard global health security.

Biofilm formation is a pivotal virulence determinant of *S. maltophilia* pathogenesis and chronic infection persistence (Magalhães et al., 2017; Isom et al., 2021). The organism’s robust biofilm-producing capability not only enhances antimicrobial tolerance but also facilitates long-term host colonization. The structured microbial communities which form biofilms establish protective matrices which can help them to evade immune surveillance and diminish antibiotic efficacy (Uberoi et al., 2024). In this context, phage therapy has demonstrated remarkable efficacy across experimental infection models, offering novel solutions for combating resistant pathogens (Visnapuu et al., 2022). Phages, defined as naturally occurring bacterial viruses, exhibit species-specific lytic activity against their target bacteria (Salmond and Fineran, 2015; Biswas et al., 2024). Unlike broad-spectrum antibiotics, their precision targeting minimizes collateral damage to the commensal microbiota (Guo et al., 2024). The self-replicating nature of phages amplifies their therapeutic potential through bacterial load-dependent amplification at infection sites. Significantly, phages demonstrate unique biofilm-penetrating capabilities—a critical advantage over conventional antibiotics that frequently fail to penetrate these protective structures (Guo et al., 2024).

Phage therapy epitomizes precision medicine advancements, enabling tailored therapeutic development against specific pathogens (Bosco et al., 2023; Guo et al., 2024). This approach has the potential to address the urgent need for novel antimicrobials, while aligning with sustainable healthcare principles through microbiome preservation. Expanding research has continued to reveal broader applications spanning infection prophylaxis, agricultural biotechnology, and environmental management, further underscoring their translational significance (Sharma et al., 2016; Pye et al., 2024).

In this context, in the present study we isolated and characterized a novel lytic phage vB_SmaS_QH16 targeting *S. maltophilia*, collected from hospital sewage. Comprehensive evaluation of this phage’s therapeutic potential included lytic efficiency profiling, host range determination, physicochemical stability assessment, and anti-biofilm capacity quantification. Through integrated *in vitro* and *in vivo* analyses, we sought to establish its pharmacodynamic profile and safety parameters, thereby laying groundwork for potential clinical translation.

## 2 Materials and methods

### 2.1 Bacterial strain collection and multilocus sequence typing analysis

We collected 73 *S. maltophilia* strains from clinical patient samples: *S. maltophilia* no. 981, isolated from a blood sample, was designated as the host strain for phage screening; the remaining 72 strains were used for subsequent host range determination experiments. All strains were routinely cultured in LB liquid medium or LB agar medium under a constant temperature of 37°C, and cryopreserved in LB broth supplemented 30% (v/v) glycerol at −80°C for future use.

Genomic DNA of the bacterial strain was extracted using a DNA extraction kit. DNA samples meeting the library construction standards were used to construct libraries with the ALFA-SEQ DNA Library Preparation Kit. These libraries were then sequenced on the Illumina NovaSeq 6000 platform with 150 bp paired-end reads. The raw sequencing data were later subjected to quality control and subsequently assembled *de novo* using SPAdes v3.13.0 software. The assembled sequences were submitted to the MLST online platform [MLST 2.0](https://cge.food.dtu.dk/services/MLST/) for sequence typing (ST) determination by aligning against seven housekeeping genes (atpD, gapA, guaA, mutM, nuoD, ppsA, recA) (Kaiser et al., 2009).

### 2.2 Phage isolation and purification

Phage vB_SmaS_QH16 was isolated and purified from sewage samples of Qinghai Provincial People’s Hospital (36.62°N, 101.78°E; altitude 2,261 m) using the double-layer agar method (Wen et al., 2022). In brief, fresh sewage was initially centrifuged at 5,000 × *g* for 5 min to remove particulate matter, followed by filtration of the supernatant through 0.22 μm membranes (Millipore, USA). Subsequently, 5 mL filtrate was mixed with an equal volume of logarithmic-phase *S. maltophilia* no.981 culture (OD_600_ = 0.4–0.6) and incubated at 37°C with 180 rpm agitation overnight for enrichment. The enriched culture was then centrifuged and filtered through 0.22 μm membranes to obtain a phage-enriched lysate. For primary screening, 100 μL of this lysate was combined with host bacterial suspension in 1.5% (w/v) LB semi-solid agar (pre-warmed to 50°C), overlaid onto LB agar plates, and incubated inverted at 37°C for 18–24 h. Discrete plaques were excised using sterile pipette tips, and eluted in phosphate-buffered saline (PBS) buffer. Serial dilutions of phage suspensions were mixed with 100 μL logarithmic-phase *S. maltophilia* no.981 culture and 5 mL 1.5% LB agar, then overlaid on fresh LB plates. After overnight incubation at 37°C, this purification cycle was repeated until homogeneous plaque morphology was achieved, ensuring phage purity.

### 2.3 Transmission electron microscopy

The morphology of the phage was observed using transmission electron microscopy (TEM). In brief, 20 µL of phage suspension (≥10^9^ PFU/mL) were applied to a copper grid, and allowed to air-dry for 10 minutes. Excess liquid was then removed using filter paper, and 20 µL of 2% phosphotungstic acid solution was added to the grid for negative staining for 5 minutes. This stain was then removed with filter paper, and the grid was air-dried at room temperature for 30 minutes. Finally, the phage morphology was visualized and photographed using a transmission electron microscope (Hitachi HT7700, Japan) at 80 kV.

### 2.4 Phage host range and proliferation analysis

The host specificity of phage vB_SmaS_QH16 was evaluated using the double-layer agar spot assay (Wang et al., 2024), as follows: 100 μL of the test bacterial suspension (OD_600_ = 0.4) was mixed thoroughly with 5 mL of preheated 1.5% LB semi-solid agar at 50°C, after which the mixture was immediately poured onto an LB solid agar plate to form a uniform double-layer substrate. After the substrate had solidified completely, 10 μL of phage lysate (titer ≈ 10^10^ PFU/mL) was spotted onto the surface of the plate. The plate was then incubated at 37°C for 12–16 hours. Results were determined based on plaque formation on the plate: “++” indicates the formation of clear and distinct phage plaques; “+” indicates weak intensity plaque formation; “−” indicates no phage plaque formation.

For bacterial strains that showed positive results in the host range assay, the phage proliferation ability was quantitatively analyzed using the efficiency of plaquing (EOP) (Abdelghafar et al., 2023). In the experiment, 100 μL of bacterial suspension in the logarithmic growth phase (OD_600_ = 0.4–0.6) was thoroughly mixed with an equal volume of phage gradient dilution (10^3^ to 10^8^ PFU/mL), and plated onto double-layer agar plates. After incubation at 37°C, the number of phage plaques was counted. The EOP value was calculated as the ratio of phage titer on the target bacterial strain to that on the host strain *S. maltophilia* no.981. The phage proliferation efficiency on different host bacteria was classified into four levels based on the EOP value: high productivity (EOP ≥ 0.5), moderate productivity (0.1 ≤ EOP < 0.5), low productivity (0.001 ≤ EOP < 0.1), and inefficient (EOP < 0.001). To ensure the reliability of the experimental results, all experiments were repeated in triplicate.

### 2.5 Determination of the optimal multiplicity of infection

The optimal multiplicity of infection (MOI), defined as the ratio of phages to host bacteria during the infection process, was determined by mixing phage vB_SmaS_QH16, *S. maltophilia* no.981 (concentration of 10^8^ CFU/mL) with different concentrations of phage vB_SmaS_QH16, with MOI values set at 100, 10, 1, 0.1, 0.01, 0.001, and 0.0001. The mixtures were incubated at 37°C with shaking at 180 rpm overnight. Following incubation, the cultures were filtered through a 0.22 μm filter membrane to remove bacterial cells, diluted to an appropriate concentration with PBS buffer, and the phage titer was determined using the double-layer plaque assay. The MOI corresponding to the highest phage titer was defined as the optimal MOI (Zhang et al., 2022). This experiment was independently performed three times to ensure reliability and reproducibility of the results.

### 2.6 One-step growth curve

The one-step growth curve experiment was performed according to a previously described method (Bai et al., 2022), with necessary optimizations based on preliminary experiments. The procedure was as follows: Phage vB_SmaS_QH16 was mixed with *S. maltophilia* no.981 (10^8^ CFU/mL) at the optimal MOI and incubated at 37°C for 10 minutes to allow adsorption. Subsequently, the mixture was centrifuged at 4°C and 10,000×*g* for 5 minutes, after which the supernatant was discarded. The pellet was washed and resuspended in PBS buffer, followed by a second centrifugation (4°C, 10,000×*g*, 5 minutes). The supernatant was then discarded again, and the washing and centrifugation steps were repeated twice to completely remove unabsorbed free phage particles. Subsequently, the pellet was resuspended in 10 mL of pre-equilibrated LB liquid medium (37°C), and incubated at 37°C with shaking at 180 rpm for 120 minutes. Starting from the beginning of the culture (t = 0), 100 μL samples were taken every 10 minutes. After sampling, the samples were immediately diluted to an appropriate concentration, and the phage titer was determined using the double-layer agar plate method. The experiment was repeated in triplicate, and the average values were used to plot the growth curve. In the one-step growth curve experiment, the latent period is defined as the time interval from phage adsorption to the onset of the first lysis; the burst phase refers to the period during which phages are rapidly released after completing replication; and the burst size is defined as the ratio of the number of progeny phages to the number of initially infected cells.

### 2.7 Assessment of physicochemical stability

To evaluate the physicochemical stability of phage vB_SmaS_QH16, its activity retention under various environmental conditions was systematically examined. In brief, phage samples were exposed to water baths at 4°C, 25°C, 37°C, 50°C, 60°C, 70°C, and 80°C for 1 hour to assess thermostability (Han et al., 2021). For pH tolerance, phage suspensions in SM buffer were adjusted to a pH gradient (1–14) and incubated at 37°C for 1 hour, followed by viability testing (Bai et al., 2022). The UV resistance was evaluated by exposing phage suspensions to UV radiation (196.67 ± 28.27 μW/cm²) at a distance of 30 cm for 60 minutes, with sampling every 10 minutes (Ahmad et al., 2024). Organic solvent tolerance was tested by incubating phage suspensions in SM buffer containing 1% (v/v) chloroform at 37°C for 1 hour (Ahmad et al., 2024). Phage titers for all treated samples were determined using the double-layer agar plate method, and all experiments were independently repeated three times.

### 2.8 Phage genome sequencing and bioinformatics analysis

The genomic DNA of phage vB_SmaS_QH16 was extracted using the QIAamp DNA Mini Kit (Qiagen, Germany), and sequencing was performed on the DNBSEQ-T7 platform with PE150 reads. Raw sequencing data were quality-controlled and filtered using fastp (v0.23.2) to obtain high-quality clean reads for use in *de novo* genome assembly with metaSPAdes (v3.15.4) (Nurk et al., 2017). Gene prediction and functional annotation were performed using Prokka (v1.14.6) (Seemann, 2014), and the annotation results were compared against the NCBI Non-redundant Protein Database (NR) using BLASTp to obtain homologous sequence information. Virulence and antibiotic resistance genes were identified using the VFDB (Virulence Factor Database) (Liu et al., 2021) and ResFinder (v4.1) (Bortolaia et al., 2020m), respectively. The phage lifestyle was predicted using the phage AI platform, version 1.0.2 (Tynecki et al., 2020). A genomic circular map was generated using the online tool Proksee (https://proksee.ca/) (Grant et al., 2023) to visualize the distribution of gene functions.

To analyze genomic relatedness, the whole-genome sequence was compared using the NCBI BLASTn algorithm to identify clusters of highly homologous phage sequences. Subsequently, the VIRIDIC online platform (https://rhea.icbm.uni-oldenburg.de/VIRIDIC/) (Moraru et al., 2020) was applied to perform average nucleotide identity (ANI) analysis of the selected sequences. The phylogenetic analysis of phage vB_SmaS_QH3 was performed using VICTOR (Meier-Kolthoff and Göker, 2017) (https://ggdc.dsmz.de/victor.php#), while its taxonomic status was validated through maximum-likelihood phylogenetic tree construction of highly conserved proteins (e.g., terminase large subunit and major capsid protein) using MEGA11 (Tamura et al., 2021)(https://www.megasoftware.net/).

### 2.9 Evaluation of *in vitro* bacterial lysogenic efficiency

The lysogenic efficiency of phage vB_SmaS_QH16 was evaluated in a dynamic antibacterial experiment (Chen et al., 2023). The experiment was set up with an MOI gradient (MOI = 100, 10, 1, 0.1, 0.01, 0.001, 0.0001), as follows: 100 μL of host bacterial suspension in the logarithmic growth phase (10^6^ CFU/mL) and 100 μL of gradient-diluted phage suspension (10^8^–10² PFU/mL) were inoculated into a 96-well plate to achieve the predetermined MOI. An equal volume of bacterial suspension with no phage infection was used as a control. The reaction system was placed in a microplate reader (Sunrise, Tecan, Switzerland) for constant-temperature shaking incubation at 37°C. The OD_600_ absorbance was automatically measured every 30 minutes for continuous monitoring over 24 hours. By analyzing the real-time growth curves, the rate of absorbance decrease relative to the control group was calculated for each MOI group to characterize the immediate lytic kinetics of the phage.

### 2.10 Impact of phage vB_SmaS_QH16 on *S. maltophilia* biofilms

#### 2.10.1 Crystal violet assay for biofilm quantification

To evaluate the inhibitory and eradication effects of phage vB_SmaS_QH16 on *S. maltophilia* no.981 biofilms, we applied a previously defined method (Soontarach et al., 2024) divided into two parts: inhibition of biofilm formation and eradication of mature biofilms. In the biofilm formation inhibition assay, 100 μL of *S. maltophilia* no.981 bacterial suspension (1×10^6^ CFU/mL) was mixed with an equal volume of phage vB_SmaS_QH16 suspension at different concentrations (10^8^, 10^7^, 10^6^, 10^5^,10^4^ PFU/mL, corresponding to MOI values of 100, 10, 1, 0.1 and 0.01, respectively). The mixtures were added to a 96-well plate and co-cultured statically at 37°C for 24 hours. In the mature biofilm eradication assay, 100 μL of *S. maltophilia* no.981 bacterial suspension (1×10^6^ CFU/mL) was first inoculated into a 96-well plate and then cultured statically at 37°C for 24 hours to form mature biofilms. Phage suspensions corresponding to different MOI values (100, 10, 1, 0.1 and 0.01) were subsequently added and incubated for an additional 24 hours. A control group containing LB broth without phage was included. After incubation, each well was gently washed three times with 300 μL of PBS to remove planktonic bacteria. Biofilms were fixed with 200 μL of methanol for 30 minutes, followed by staining with 200 μL of 0.1% crystal violet solution for 20 minutes. The wells were subsequently washed three times with PBS to remove any unbound dye. Finally, 250 μL of 33% acetic acid was added to dissolve the bound dye, after which the absorbance was measured at 570 nm using a microplate reader. To ensure the accuracy of the results, all experiments were performed in triplicate and independently repeated three times.

#### 2.10.2 MTT assay for biofilm metabolic activity

The MTT assay was performed based on established protocols (Ghasemi et al., 2021), with modifications, after the biofilm cultivation procedures described previously. Following incubation, biofilm-containing wells were washed three times with PBS to remove planktonic bacteria. Subsequently, 20 μL of MTT working solution (5 mg/mL, Shanghai Yuanye Bio-Technology Co., Ltd., China) and 200 μL fresh LB medium were added to each well, followed by 3-hour incubation at 37°C in darkness. The supernatant was then carefully removed, and 200 μL dimethyl sulfoxide (DMSO) was added to dissolve then formazan crystals under low-speed vortexing for 20 min. The absorbance was measured at 492 nm using a microplate reader. All assays included triplicate technical replicates with three independent biological repetitions.

#### 2.10.3 Fluorescence microscopy of biofilm architecture

Biofilms grown in 6-well plates according to the aforementioned protocol were gently washed with PBS to eliminate non-adherent cells. These were then fixed with 4% paraformaldehyde for 15 min, after which the samples were rinsed and air-dried. Biofilms were then stained with 0.02% acridine orange solution under light-protected conditions for 15 min, followed by PBS washes to remove unbound dye. After dark-drying, biofilm structures were visualized using fluorescence microscopy (excitation 488 nm) (Wang et al., 2024).

### 2.11 Evaluation of phage vB_SmaS_QH16 efficacy in *Galleria mellonella* larvae

*G. mellonella* larvae (provided by Huide Biotechnology Co., Ltd., Tianjin, China) were used as an animal model to evaluate the *in vivo* anti-infective efficacy of the target phage (Li et al., 2023). Healthy fifth-instar larvae (weight: 273 ± 31 mg, length: 2.72 ± 0.13 cm) were selected, with 10 larvae per group. Before injection, the injection site was disinfected with a swab containing 75% ethanol. The host bacteria (*S. maltophilia* no.981) were cultured to the logarithmic growth phase, washed three times with PBS, and resuspended to the desired concentration. To determine the appropriate infectious dose, 10 μL of bacterial solution at different concentrations (1×10^5^, 1×10^6^, 1×10^7^, 1×10^8^, and 1×10^9^ CFU/mL) was injected into the posterior left leg of the larvae using a micro-injector (Gaoge, Shanghai, China). Following injection, the larvae were placed in petri dishes lined with sterile filter paper, and incubated at 37°C and 70% humidity in the dark. The survival status of the larvae was observed and recorded every 8 hours for up to 72 hours to assess the impact of different infectious doses on larval survival. *G. mellonella* larvae were considered dead when they showed no response to touch (Wei et al., 2016).

To evaluate the therapeutic effect of the phage, larvae were initially infected with bacteria, and then treated with phage injections (Li et al., 2023). *S. maltophilia* no.981 was subsequently resuspended and diluted to 1×10^8^ CFU/mL, after which 10 µl of cell resuspension was injected into the posterior left leg. One hour post-infection, 10 μL of phage suspension at different MOI values (100, 10, 1, and 0.1) was injected into the opposite side of the bacterial injection site. The larvae were then placed in petri dishes with sterile filter paper, and incubated at 37°C and 70% humidity in the dark. Survival status was recorded every 8 hours for 72 hours to assess the therapeutic effects of different phage doses.

In the prophylactic phage treatment model, the phage was injected into *G. mellonella* larvae 1 hour prior to bacterial infection (Li et al., 2023). In brief, 10 μL of phage suspension at different MOI values (100, 10, 1, and 0.1) was injected into the posterior left leg of the larvae. After 1 hour of incubation, 10 μL of *S. maltophilia* no.981 (1×10^8^ CFU/mL) was injected into the opposite side of the phage injection site. The larvae were then placed in petri dishes with sterile filter paper and incubated at 37°C and 70% humidity in the dark. Survival status was recorded every 8 hours for 72 hours to evaluate the prophylactic effects of different phage doses. Positive control larvae were infected with *S. maltophilia* no.981 and treated with PBS. Negative controls included larvae injected with PBS alone and those injected with phage at an MOI of 100 without bacterial challenge. All experiments were repeated in triplicate.

### 2.12 Therapeutic efficacy of phage vB_SmaS_QH16 in a mouse model of infection

The mouse infection model of *S. maltophilia* no.981 was established as per literature methods (Han et al., 2022). The logarithmic growth phase bacteria were harvested, washed twice with PBS, and adjusted to a concentration of 5×10^8^ CFU/mL. In this study, SPF BALB/c mice (3–5 weeks old, 15–19 grams) were purchased from Beijing Weilitonghua Laboratory Animal Technology Co., Ltd. The Ethics Committee of Qinghai Provincial People’s Hospital approved the animal study protocol (2024-047-01). All mice received an intraperitoneal injection of cyclophosphamide (125 mg/kg) on day 4 and an intraperitoneal injection of cyclophosphamide (125 mg/kg) and dexamethasone (12.5 mg/kg) on day 1 before infection to induce immunosuppression.

Immunocompromised mice were randomly divided into six groups (13 mice per group): Group I (negative control), Group II (positive control), and treatment groups (Group III–VI). Details are as follows: Group I mice received 40 μL of PBS intranasally, followed by intranasal administration of phages (2×10^8^ PFU/mouse) 6 hours later; Group II mice were infected intranasally with 2×10^7^ CFU/mouse of bacterial suspension and then given 40 μL of PBS intranasally 6 hours post - infection (hpi); Group III–VI mice were also infected intranasally with 2×10^7^ CFU/mouse of bacterial suspension, and 6 hpi, they received phages intranasally at different MOIs: Group III, 2×10^8^ PFU/mouse (MOI=10); Group IV, 2×10^7^ PFU/mouse (MOI=1); Group V, 2×10^6^ PFU/mouse (MOI=0.1); Group VI, 2×10^5^ PFU/mouse (MOI=0.01). Mouse survival was monitored daily for 7 days. At 30 hours post-phage treatment, three mice were randomly selected from each group. Their lung tissues were collected. One part was fixed for H&E staining to evaluate histopathological changes, and the other part was homogenized and plated to quantify viable bacteria (Pu et al., 2022).

### 2.13 Statistical analysis

All data were analyzed using GraphPad Prism 10.1.2. Continuous data are presented as the mean ± SD. For comparisons among multiple groups, one-way analysis of variance (ANOVA) followed by Dunnett’s multiple comparison test was performed to assess intergroup differences. Survival curves were constructed using the Kaplan-Meier method, and differences in survival rates between groups were evaluated using the Log-rank (Mantel-Cox) test. Statistical significance was defined as *P* < 0.05, with significance levels indicated as follows: NS not significant, *P < 0.05, **P < 0.01, ***P < 0.001, and ****P < 0.0001.

## 3 Results

### 3.1 Isolation and morphological characterization of phage vB_SmaS_QH16

**Figure 1:**
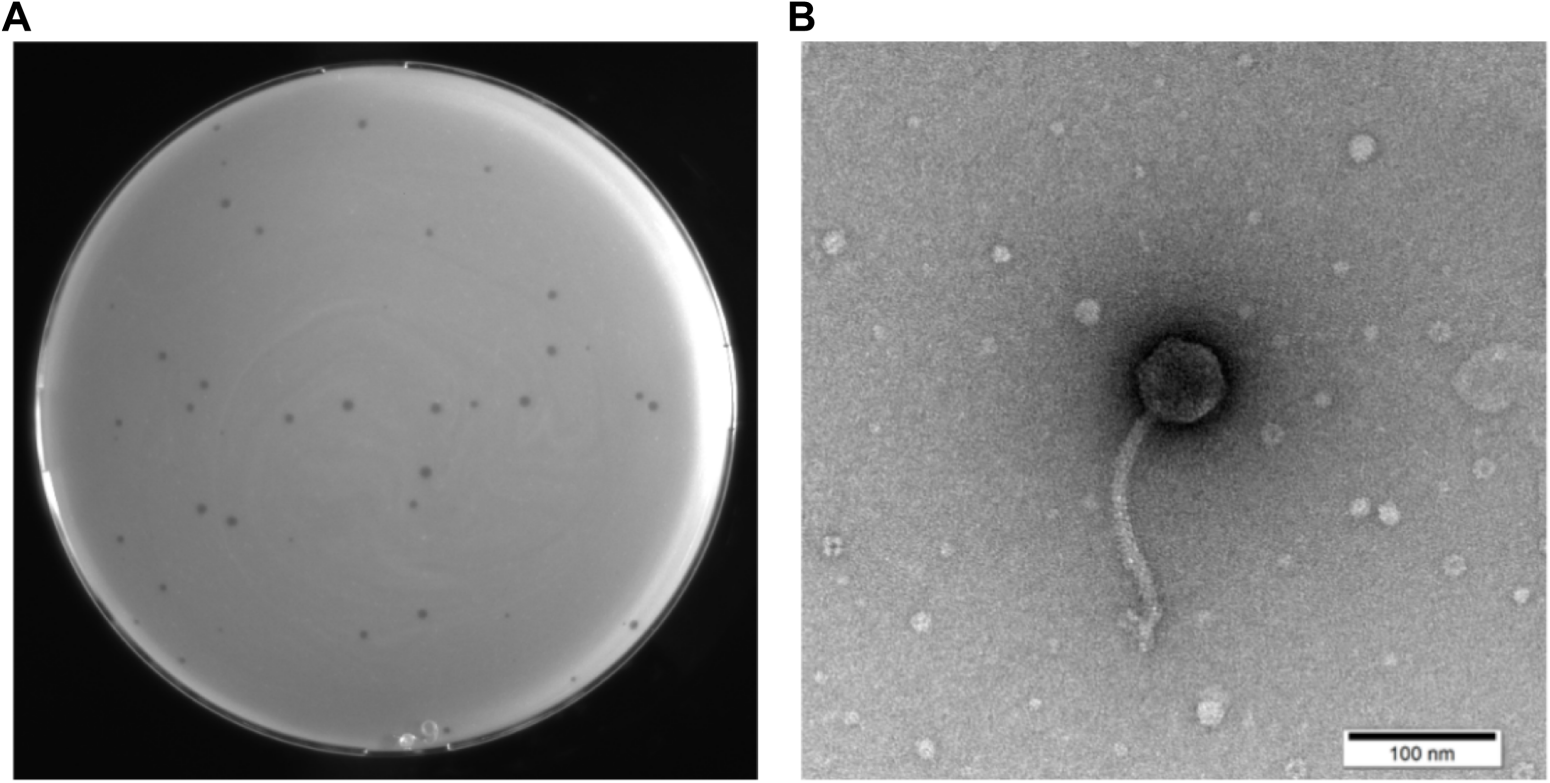
Morphological characterization of phage vB_SmaS_QH16. **(A)** Image of the transparent plaques formed by the phage on a lawn of the host bacterium *S. maltophilia* no.981; **(B)** Transmission electron micrograph of the phage (scale bar, 100 nm).

A novel phage was successfully isolated from a hospital sewage sample using *S. maltophilia* no.981 as the host bacterium. On double-layer agar plates, this phage formed transparent plaques with a diameter of 1 to 2 mm (**Figure 1A**), featuring clear edges and no “halo” effect, potentially indicative of a lytic life cycle. TEM observations revealed that phage vB_SmaS_QH16 exhibited typical morphological characteristics of tailed phages: the head was icosahedrally symmetrical with a diameter of 54.36 ± 1.68 nm, while the tail constituted a non-contractile, elongated tube with a length of 166.97 ± 6.37 nm, terminating in several tail fibers (**Figure 1B**). Based on the morphological characteristics and the 2024 ICTV classification criteria, vB_SmaS_QH16 was classified within the class Caudoviricetes (NCBI:txid3237192).

### 3.2 MLST analysis, host range, and EOP assay

MLST analysis of 73 *S. maltophilia* strains revealed 28 distinct sequence types (STs), with 21 strains not matching any specific ST, indicating at least 29 distinct lineages, and highlighting high genetic diversity (**Table 1** and **Supplementary Table 1**). Phage vB_SmaS_QH16 lysed 35 strains (47.95%), covering 13 STs (ST4, ST8, ST115, ST138, ST185, ST208, ST212, ST223, ST365, ST517, ST518, ST644, ST1139) and 13 untyped strains. The EOP results showed significant variation in lysis efficiency, with EOP values ranging from <0.001 to 1.375 ± 0.163. Classification revealed 12 strains (16.44%) as high-producers (EOP ≥ 0.5), 4 (5.48%) as moderate-producers (0.1 ≤ EOP < 0.5), 1 (1.37%) as low-producers (0.001 ≤ EOP < 0.1), and 18 (24.66%) as inefficient (EOP < 0.001). Notably, three strains (4.11%) exhibited an EOP > 1, with the highest showing a 37.5% higher plaque efficiency than the host *S. maltophilia* no.981, indicating superior proliferation in the target host.

**Table 1:** Analysis of phage vB_SmaS_QH16 host range and proliferation capacity.

Stain no.	Isolation year	Specimen source	MLST type	Host range reactivity	EOP value	EOP level
2831	2024	Sputum	4	−		
2923	2024	Sputum		+	<0.001	Inefficient
3738	2024	Sputum		−		
3135	2024	Sputum	517	−		
2964	2024	Sputum	185	+	<0.001	Inefficient
3589	2024	Sputum	233	−		
4431	2024	Sputum	27	−		
462a	2018	Blood	644	+	<0.001	Inefficient
981	2019	Blood		++	1	High
935a	2018	Blood	644	+	<0.001	Inefficient
423a	2018	Blood		++	0.771 ± 0.132	High
441a	2016	Blood		++	1.102 ± 0.154	High
450	2016	Blood		++	0.963 ± 0.188	High
5662	2024	Sputum	4	−		
5808	2024	urine	223	++	0.596 ± 0.055	High
8000	2024	Sputum	583	−		
8445	2024	Joint effusion	4	−		
8347	2024	Sputum	321	−		
2579	2024	Sputum	152	−		
1803	2023	Sputum	212	++	<0.001	Inefficient
2552	2024	Sputum	162	−		
2459	2024	Sputum	115	++	0.027 ± 0.029	LOW
2397	2024	Sputum	4	−		
2463	2024	Sputum	84	−		
0815	2024	Sputum	4	+	<0.001	Inefficient
5978	2024	Sputum	887	−		
3798	2024	Sputum		−		
3593	2024	Sputum	208	++	0.795 ± 0.895	High
3309	2024	Sputum	31	−		
5282	2024	Sputum	152	−		
3873	2024	Sputum		++	0.171 ± 0.076	Medium
6359	2024	Sputum	84	−		
5818	2024	Sputum	4	−		
6261	2024	Sputum		+	<0.001	Inefficient
5790	2024	Sputum		+	0.645 ± 0.504	High
3595	2024	Sputum	208	−		
7043	2024	Sputum	4	−		
7596	2024	Sputum	1139	++	0.177 ± 0.087	Medium
361	2018	Blood		−		
3751	2024	Sputum		−		
853	2014	Blood	517	+	<0.001	Inefficient
2467	2024	Sputum	138	+	<0.001	Inefficient
4709	2024	Sputum	1032	−		
3573	2024	Sputum	138	−		
1866	2024	Balf	138	++	<0.001	Inefficient
0512	2024	Sputum	365	++	0.926 ± 0.085	High
187	2023	Sputum	845	−		
508	2023	Sputum	212	+	<0.001	Inefficient
1599	2023	Balf	309	−		
746	2023	Balf	1134	−		
1078	2023	Sputum	53	−		
7049	2024	Balf	1139	++	0.255 ± 0.199	Medium
727	2019	Bile		−		
6179	2023	Pleural effusion		−		
047	2019	Sputum		++	<0.001	Inefficient
107	2023	Blood	518	++	1.261 ± 0.217	High
0600	2022	Balf	8	+	<0.001	Inefficient
0154	2024	Sputum	84	−		
935	2018	Blood	644	+	<0.001	Inefficient
5997	2023	Pleural effusion		−		
811	2018	Blood	644	+	<0.001	Inefficient
462	2018	Blood	644	+	<0.001	Inefficient
915	2017	Balf		−		
423	2019	Drainage fluid		++	0.309 ± 0.129	Medium
013	2018	Blood		++	0.831 ± 0.053	High
441	2016	Blood		++	1.375 ± 0.163	High
550	2016	Blood		++	0.693 ± 0.252	High
564	2023	Sputum	249	−		
2991	2024	Sputum	138	++	<0.001	Inefficient
2947	2024	Sputum	138	+	<0.001	Inefficient
1509	2022	Balf	309	−		
3345	2024	Sputum	152	−		
3971	2024	Sputum	4	−		

### 3.3 MOI and one-step growth curve

**Figure 2:**
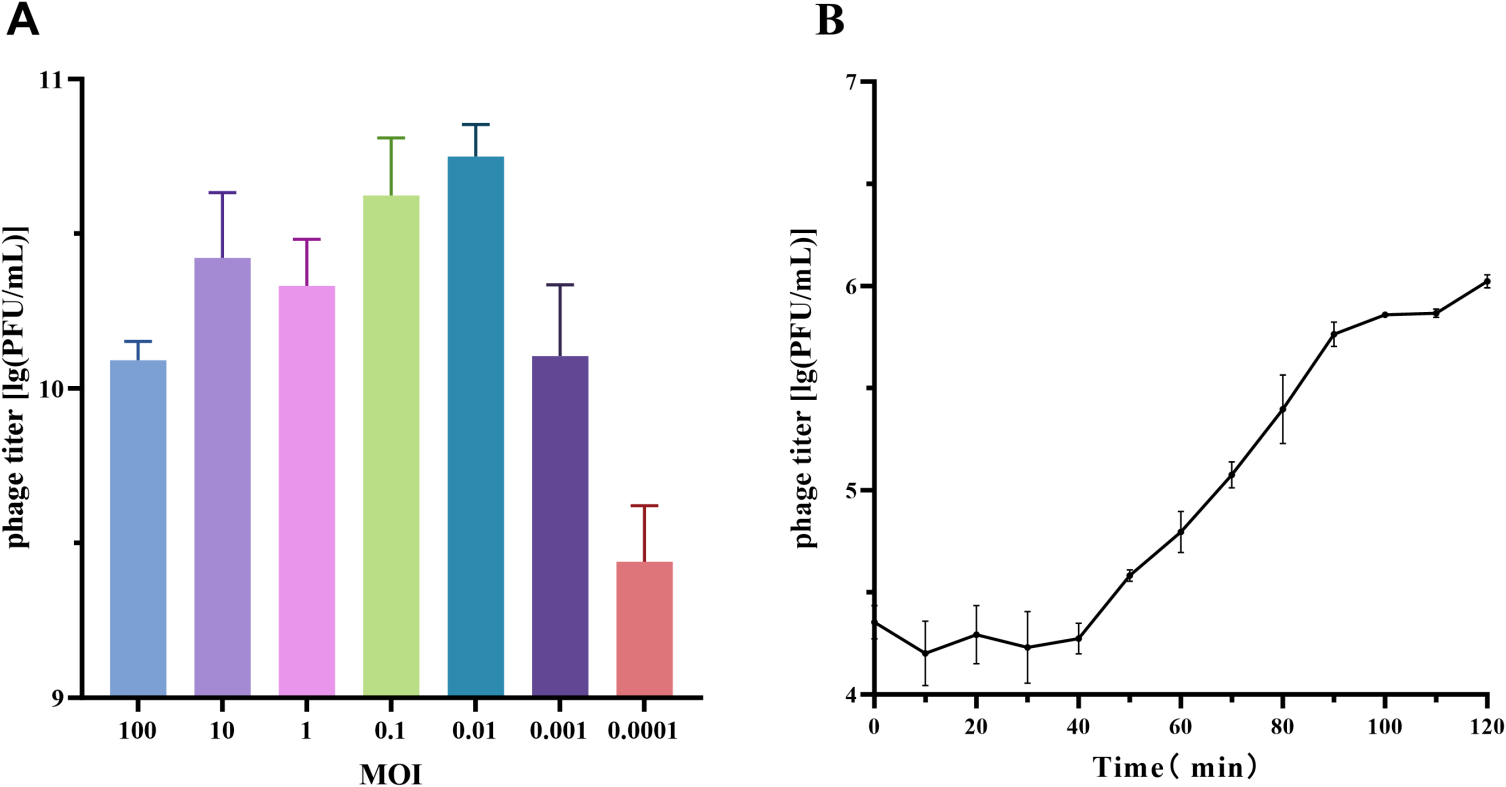
Growth characteristics of phage vB_SmaS_QH16. **(A)** Optimal MOI determination and **(B)** one-step growth curve of phage vB_SmaS_QH16 at an MOI of 0.01.

In the MOI experiment for phage vB_SmaS_QH16, the highest progeny phage titer was achieved at an MOI of 0.01, reaching 5.73×10^10^ PFU/mL. Thus, the optimal MOI for phage vB_SmaS_QH16 was determined to be 0.01 (**Figure 2A**).

For the one-step growth curve at this MOI, the phage titer remained stable, with no significant changes during the first 40 minutes post-infection. However, from 40 to 100 minutes, the titer increased rapidly, indicating active phage replication and release. The latent period was approximately 40 minutes, while the lysis phase lasted 60 minutes, with an average burst size of approximately 37.69 PFU/cell (**Figure 2B**).

### 3.4 Stability under physicochemical conditions

**Figure 3:**
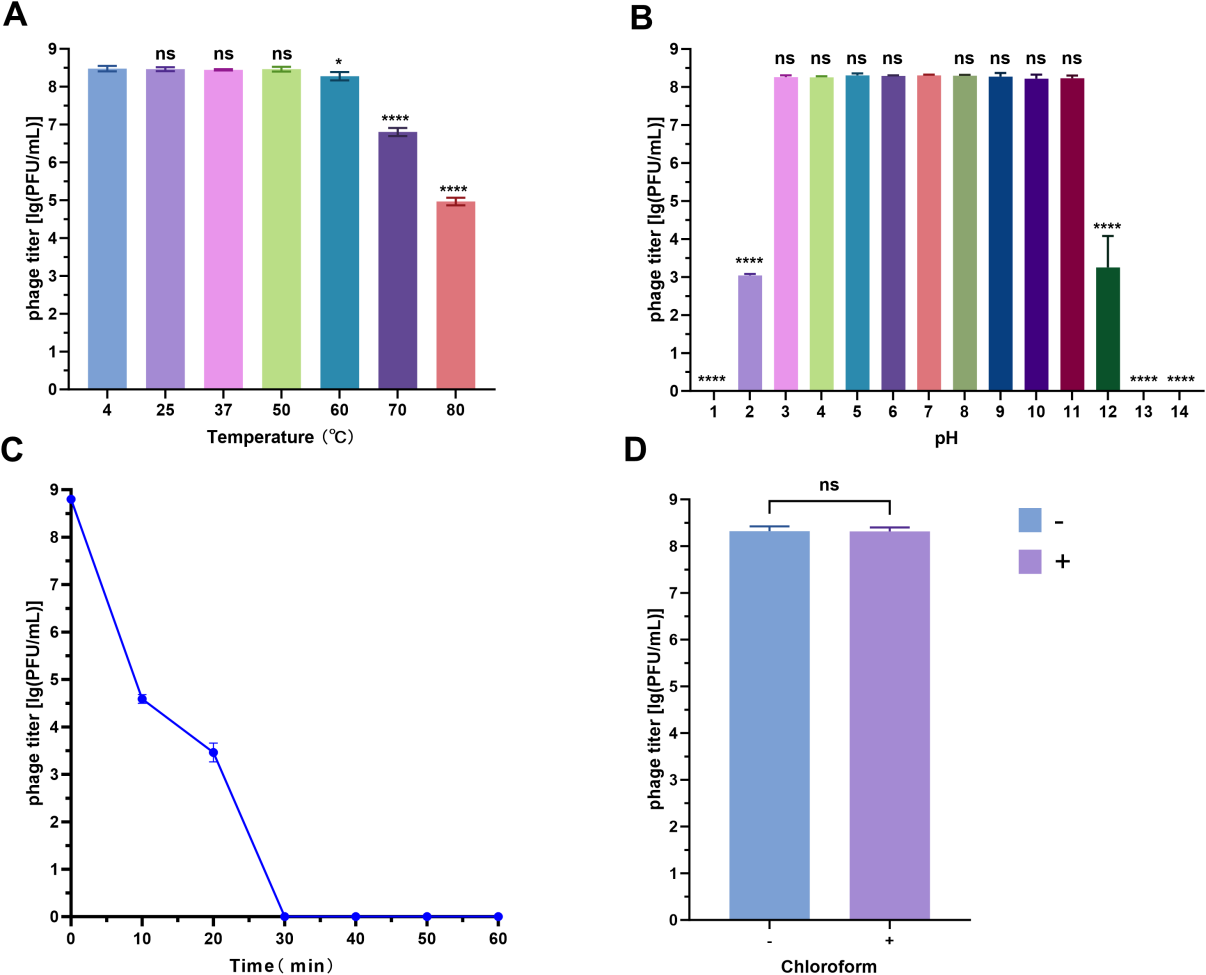
Stability analysis of phage vB_SmaS_QH16 under different physicochemical conditions. **(A)** Thermal tolerance (4–80°C); **(B)** pH adaptability (1–14); **(C)** UV resistance (0–60 min exposure); **(D)** Chloroform resistance analysis (0% vs 1% v/v). Data are presented as the mean ± SD (n = 3). Statistical significance in panels **(A, B, D)** was determined by one-way ANOVA (ns, not significant,*P < 0.05; **P < 0.0001).

Phage vB_SmaS_QH16 exhibited specific stability across various physicochemical conditions. Thermostability testing revealed no significant titer change (*P* > 0.05) after 1 hour of incubation at 4 to 50°C, with peak activity retained. Although the titer gradually decreased at higher temperatures, it only dropped by 3 log10 at 80°C, indicating good thermal stability (**Figure 3A**). In pH tolerance experiments (**Figure 3B**), the phage retained high activity between pH 3.0 and 11.0, with significant titer reductions at pH 2 and 12 (*P* < 0.0001), and complete inactivation at extreme pH values of 1, 13, and 14. The phage was highly sensitive to UV radiation, with significant inactivation after 10 minutes, and nearly complete loss of activity after 30 minutes (**Figure 3C**). Chloroform treatment had no significant effect on phage titer, suggesting minimal or no lipid content in the phage capsid (**Figure 3D**) (Mäntynen et al., 2019).

### 3.5 Genome sequencing and analysis

**Figure 4:**
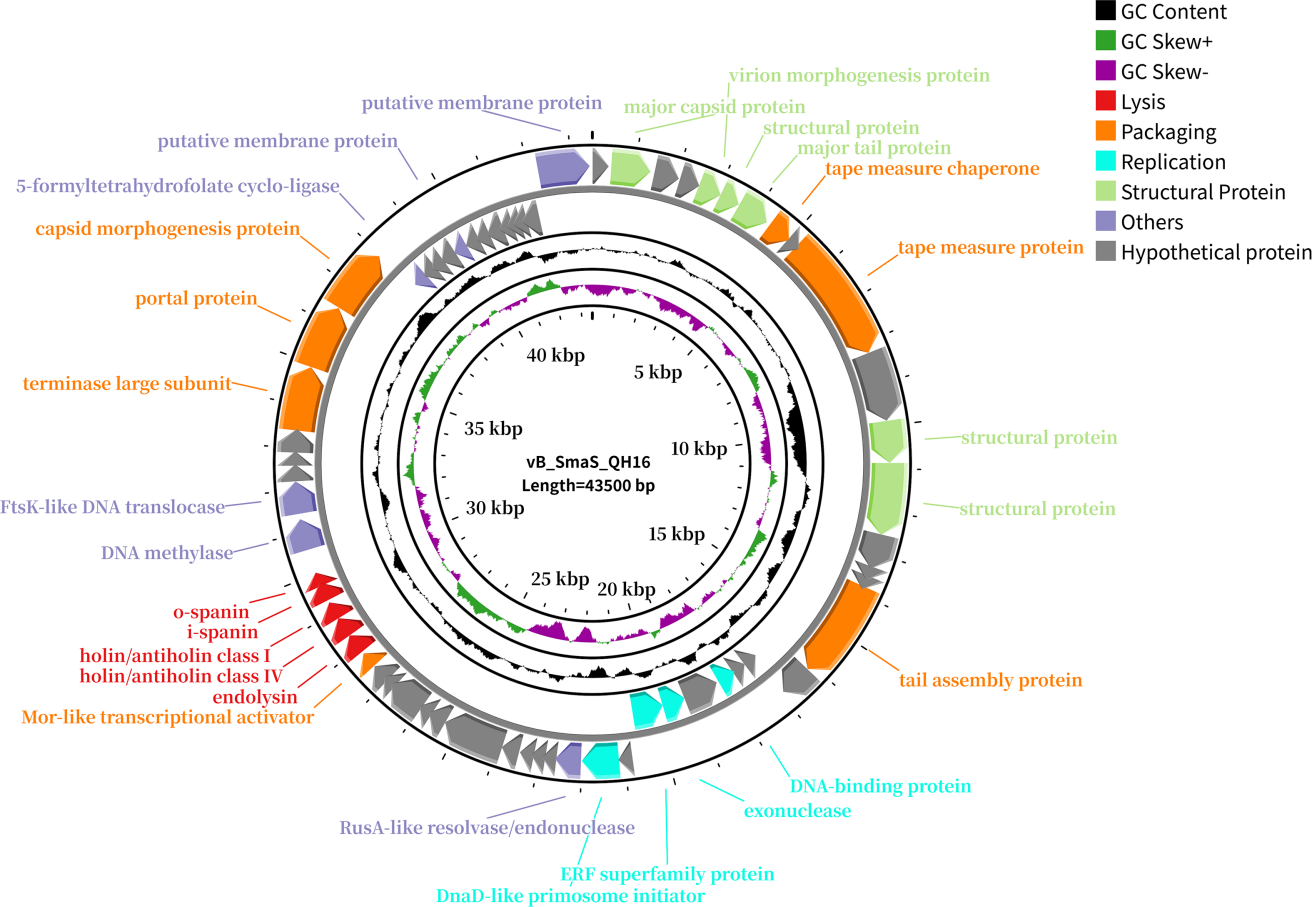
Genome map of phage vB_SmaS_QH16. All predicted ORFs are indicated by arrows, with the forward direction in the outer ring and the reverse direction in the inner ring. Colors denote functional categories: red, lysis; orange, packaging; blue, replication; green, structural proteins; purple, others; gray, hypothetical proteins.

The genome of phage vB_SmaS_QH16 (GeneBank accession number: PP921744) comprised a linear dsDNA molecule of 43,500 bp with a GC content of 67.49%. A total of 64 ORFs were predicted, with a coding density of 95.93%, while no tRNA genes were detected (**Supplementary Table 2**). Of these ORFs, 46 were on the forward strand and 18 on the reverse strand. In total, fifty-one ORFs use ATG as the start codon, while 13 use GTG. The total length of all ORFs reached 41,730 bp, averaging approximately 652 bp each, accounting for 95.93% of the entire genome. No tRNA, virulence, antibiotic resistance, or integrase genes were predicted in the vB_SmaS_QH16 genome (**Figure 4**). Structural Proteins, Replication, Packaging, Lysis, and otFunctional annotation of the ORFs against non-redundant protein databases using BLASTp revealed that these ORFs could be broadly classified into five functional modules: hers. Of the 64 ORFs, 36 are hypothetical proteins, while the remaining 28 are functional genes. Specifically, 9.34% (6/64) of the ORFs encode structural proteins essential for phage morphology and assembly, such as the major capsid protein (ORF2), virion morphogenesis protein (ORF5), major tail protein (ORF7), and additional structural proteins (ORF6, ORF12, ORF13). Overall, 6.25% (4/64) of the ORFs are involved in phage replication and DNA processing, including the DNA-binding protein (ORF21), exonuclease (ORF23), ERF superfamily protein (ORF24), and DnaD-like primosome initiator (ORF26). 10.93% (7/64) of the ORFs are associated with DNA packaging, tail and capsid assembly, such as the tape measure chaperone (ORF8), tape measure protein (ORF10), tail assembly protein (ORF17), Mor-like transcriptional activator (ORF38), terminase large subunit (ORF49), portal protein (ORF50), and capsid morphogenesis protein (ORF51). A further 7.81% (5/64) of the ORFs are involved in host lysis, including endolysin (ORF39), holin/antiholin class IV (ORF40), holin/antiholin class I (ORF41), i-spanin (ORF42), and o-spanin (ORF43). Finally, 9.38% (6/64) of the ORFs have functions that are not fully understood, or may participate in multiple stages of the phage life cycle, such as RusA-like resolvase/endonuclease (ORF27), DNA methylase (ORF44), FtsK-like DNA translocase (ORF45), 5-formyltetrahydrofolate cyclo-ligase (ORF52), and putative membrane proteins (ORF56, ORF64). Additionally, there are 37 hypothetical proteins whose functions remain unclear, requiring further research to uncover their potential importance.

### 3.6 Comparative genomics and phylogenetic analysis

**Figure 5:**
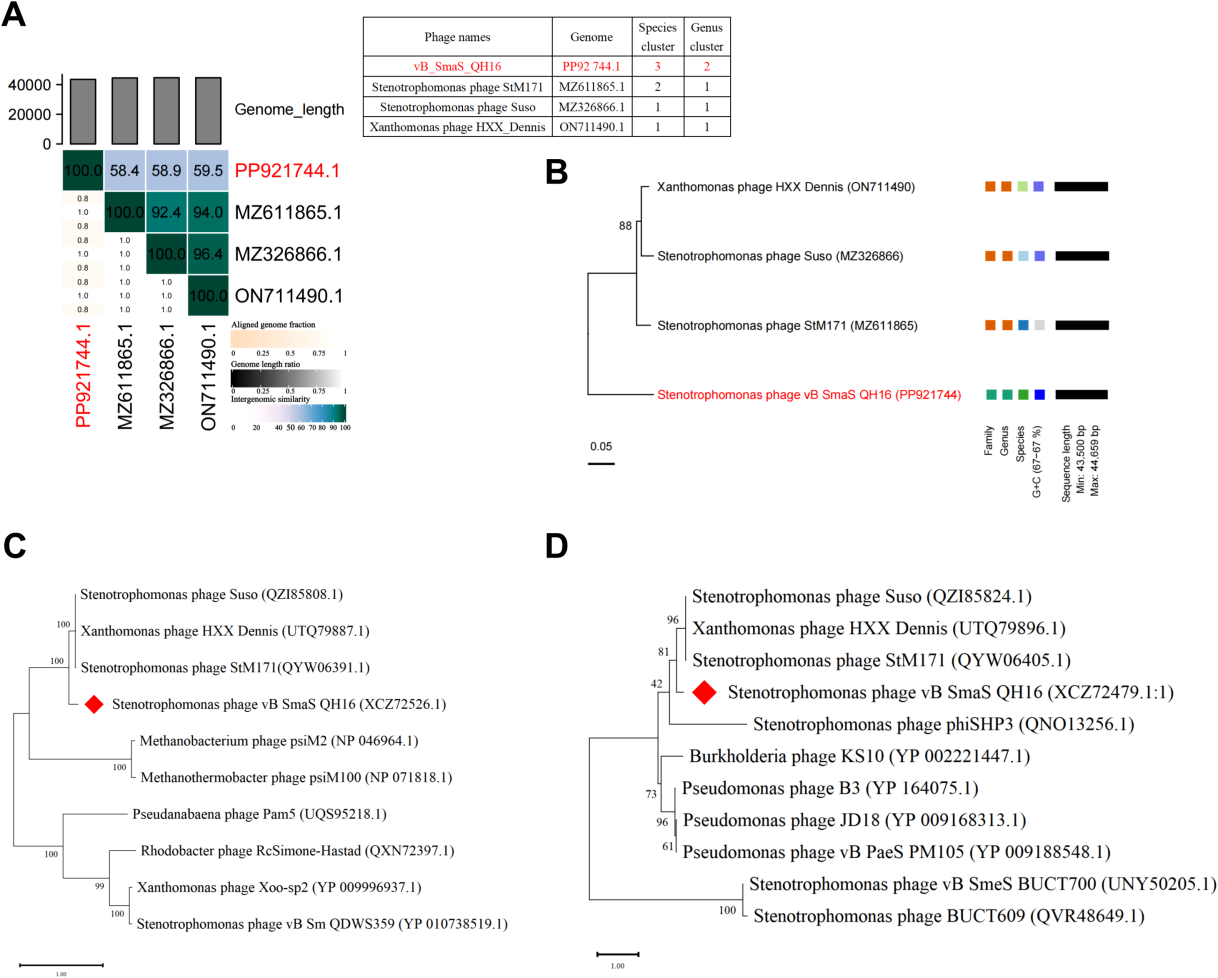
Comparative genomics and phylogenetic analysis. **(A)** VIRIDIC heatmap analysis of comparative genomic similarity; **(B)** Phylogenetic tree constructed using whole-genome VICTOR analysis; **(C)** Phylogenetic tree of the terminase large subunit; **(D)** Phylogenetic tree of the major capsid protein. The phylogenetic trees were constructed using the Maximum Likelihood (ML) method and evaluated with 100 bootstrap replicates using MEGA 11 software.

Whole-genome alignment revealed that phage vB_SmaS_QH16 shares 52–53% sequence coverage with closely related phages StM171 (MZ611865.1), HXX_Dennis (ON711490.1), and Suso (MZ326866.1) (BLASTn, E-value = 0) (**Supplementary Table 3**). However, its maximum similarity was only 79.37%, far below the species-level classification threshold (95% ANI), suggesting that it likely represents a novel species. Further analysis using VIRIDIC for ANI calculations revealed that the ANI value between vB_SmaS_QH16 and its closest relative, HXX_Dennis, was only 59.5% (**Figure 5A**), well below the genus-level threshold of 70% ANI (ICTV 2023), indicating the need to classify this phage as a new species within a novel genus.

Phylogenetic analysis using the VICTOR tool demonstrated significant differences in tree topology stability across different distance formulas (D0, D4, D6). The D4 formula yielded the highest branch support (average 88%), outperforming D0 (50%) and D6 (53%). Thus, D4 was selected for further analysis. OPTSIL clustering further identified four species clusters, two genus clusters, and two family clusters (**Figure 5B**). The phylogenetic tree showed that vB_SmaS_QH16 forms a distinct evolutionary branch, supporting its differentiation at the family, genus, and species levels. This further reinforces its classification as a member of a new genus within the class Caudoviricetes. Additionally, single-gene phylogenetic trees for the terminase large subunit (**Figure 5C**) and major capsid protein (**Figure 5D**) confirmed that vB_SmaS_QH16 is most closely related to phages StM171 (MZ611865.1), HXX_Dennis (ON711490.1), and Suso (MZ326866.1) within a larger clade. In summary, vB_SmaS_QH16 is a novel phage that can be classified within the class Caudoviricetes.

### 3.7 Effects of vB_SmaS_QH16 on biofilms

**Figure 6:**
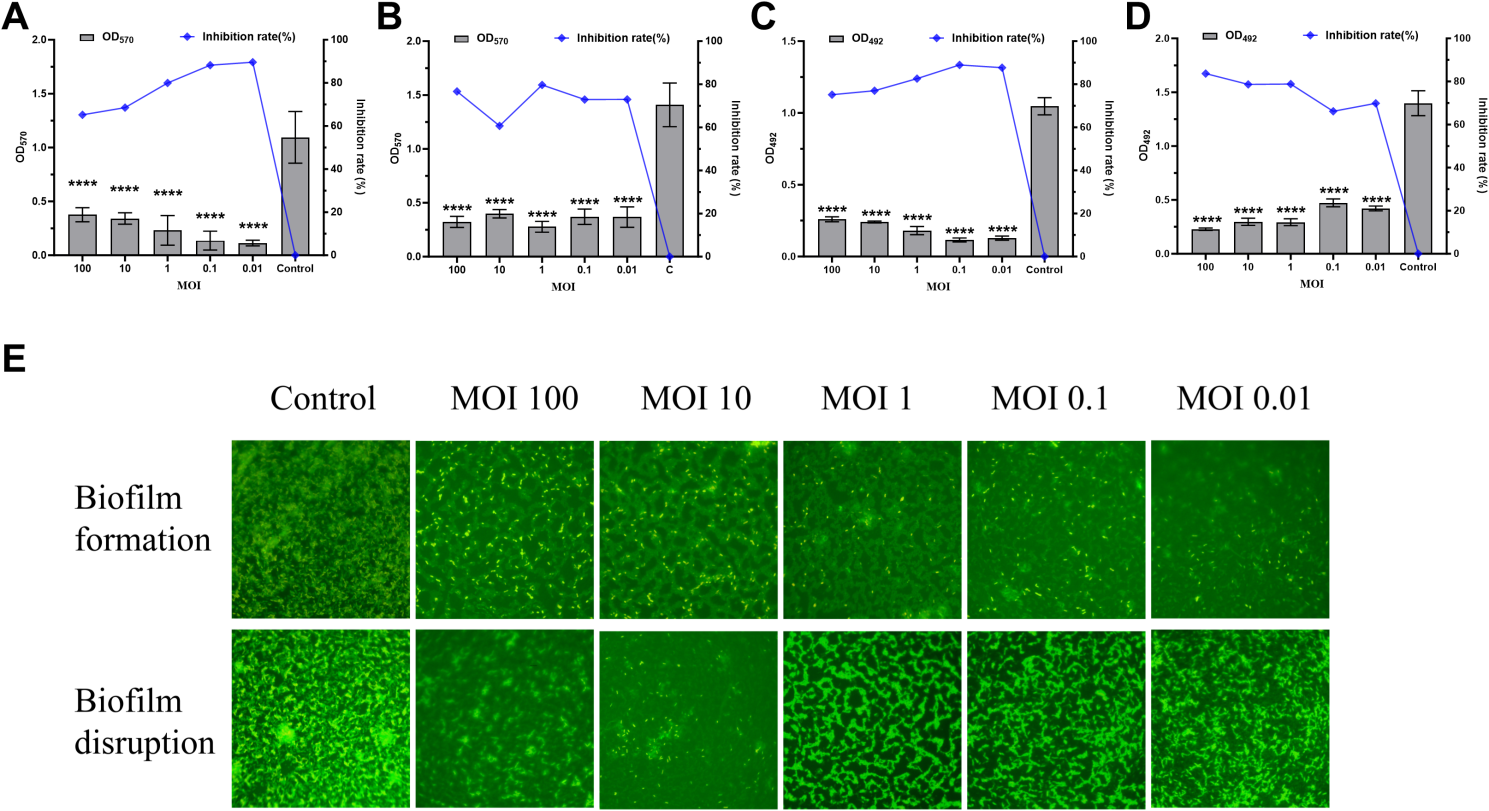
Effects of Phage vB_SmaS_QH16 on Biofilm Formation and Eradication. **(A)** Biofilm formation inhibition: Semi-quantitative analysis of the 24-hour biofilm biomass using crystal violet staining; **(B)** Mature biofilm eradication: Evaluation of residual 24-hour biofilm biomass using crystal violet staining; **(C)** Biofilm metabolic activity monitoring: Bacterial viability during biofilm formation assessed by MTT assay after 24 hours; **(D)** Efficacy of biofilm eradication: Quantification of bacterial survival rate changes after phage treatment for 24 hours using the MTT method; **(E)** Biofilm structure observation: Visualized using fluorescence microscopy (1000×) at an excitation wavelength of 488 nm after acridine orange staining.

Crystal violet staining, MTT assays, and acridine orange fluorescence staining were conducted to evaluate the total biofilm mass, metabolic activity, and three-dimensional structure, respectively, to investigate the effects of phage vB_SmaS_QH16 on *S. maltophilia* no.981 biofilms. During biofilm formation, phage treatment significantly reduced biofilm mass (*P* < 0.0001). The inhibition rate revealed a concentration-dependent increase as the MOI decreased from 100 to 0.01, rising from 65.02% to 89.61%, indicating better inhibition at lower phage concentrations (**Figure 6A**). This trend was confirmed by MTT assays, in which metabolic activity inhibition correlated with biofilm mass reduction across the different MOI groups (**Figure 6C**). Fluorescence microscopy further showed that the control group had dense three-dimensional biofilm structures, whereas the treated groups exhibited significant structural disintegration, with mesh-like or discrete distributions and a marked reduction in bacterial aggregation (**Figure 6E**). In experiments targeting mature biofilms, phage treatment led to a significant reduction in biofilm mass (*P* < 0.0001) (**Figure 6B**), with MTT results confirming a corresponding decrease in metabolic activity that positively correlated with phage concentration (**Figure 6D**). Structural analysis further revealed that treated biofilms exhibited disrupted three-dimensional architecture, forming loose, net-like morphologies with reduced bacterial density (**Figure 6E**). Overall, vB_SmaS_QH16 demonstrated significant efficacy at inhibiting biofilm formation and eradicating mature biofilms, with distinct concentration-dependent effects: lower concentrations were more effective at preventing biofilm formation, while higher concentrations were better suited to clearing established biofilms.

### 3.8 *In vitro* antibacterial activity

**Figure 7:**
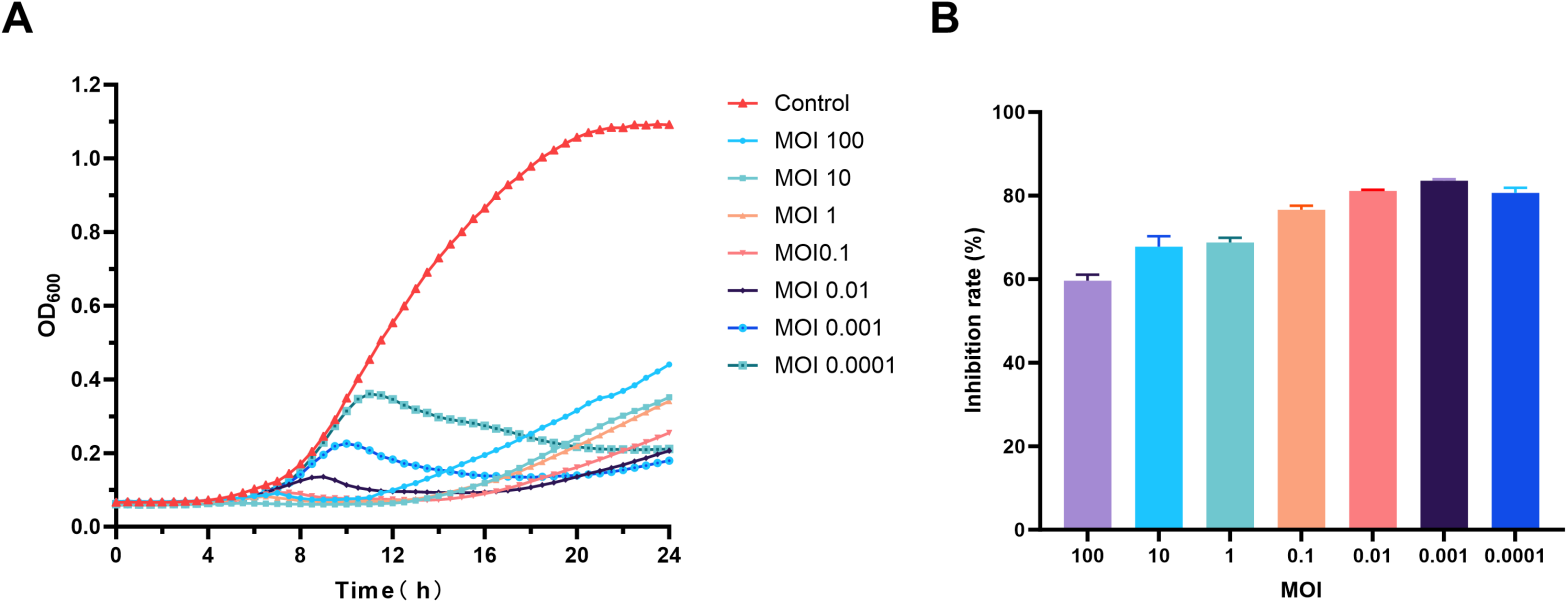
Evaluation of the *in vitro* antibacterial activity of phage vB_SmaS_QH16. **(A)** Time-bacteriostatic dynamics curve; **(B)** Inhibition rate of phage vB_SmaS_QH16 at different concentrations over 24 hours.

As shown in **Figure 7A**, the growth curve of the *S. maltophilia* no.981 control group in LB liquid medium exhibited a typical “S” shape. Following the addition of phage vB_SmaS_QH16, all phage-treated groups demonstrated significant antibacterial effects, with inhibition rates at 24 hours of 59.61% (MOI 100), 67.77% (MOI 10), 68.77% (MOI 1), 76.63% (MOI 0.1), 81.12% (MOI 0.01), 83.56% (MOI 0.001), and 80.66% (MOI 0.0001) (**Figure 7B**). However, the different concentration groups exhibited dynamic heterogeneous growth patterns: in the high-concentration groups (MOI 100–0.1), bacterial growth was completely suppressed in the initial stage (0–10 hours), indicating phage dominance. However, after 10 hours, a gradual increase in bacterial concentration was observed across the groups, indicating the emergence and proliferation of phage-resistant mutants. In the low-concentration groups (MOI 0.01–0.0001), a transient increase in bacterial concentration occurred during the initial stage (0–9 hours), reflecting a temporary advantage of the host bacteria. Subsequently, between 9 and 11 hours, an inflection point in concentration was observed, marked by a significant decrease in bacterial load, indicating that the phage established a secondary infection advantage through the lytic cycle. Notably, all treated groups began to show an increase in bacterial concentrations after 16 hours, suggesting a dynamic balance between resistant mutants and residual phages, forming a periodic pattern of “resistance escape-phage inhibition.”

### 3.9 The bacteriostatic activity of vB_SmaS_QH16 in *G. mellonella*

**Figure 8:**
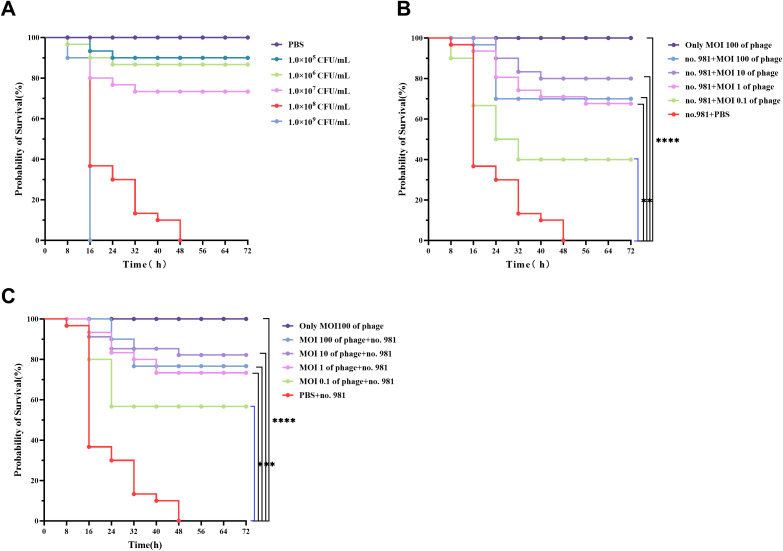
*In Vivo* bactericidal effects of vB_SmaS_QH16 in *G. mellonella* larvae. **(A)** Survival curves of larvae after injection of different concentrations of *S. maltophilia* no.981; **(B)** Survival curves of infected *G. mellonella* larvae treated with phage vB_SmaS_QH16; **(C)** Survival curves of larvae protected by phage vB_SmaS_QH16. Survival rates of *G. mellonella* larvae were analyzed using the Kaplan-Meier method, while statistical analysis was performed using the Log-rank (Mantel-Cox) test. A *P*-value less than 0.05 was considered statistically significant. Symbols are defined as follows: ***P < 0.001; ****P < 0.0001.

To assay bacteriostatic activity, a gradient concentration of host bacteria (1×10^5^–1×10^9^ CFU/mL) was injected to determine the optimal bacterial concentration for the *G. mellonella* infection model (**Supplementary Figure 1**). The results revealed a significant positive correlation between the lethal effect of the host bacteria and the concentration. The group injected with 1×10^9^ CFU/mL exhibited 100% mortality within 16 hours; however, this rapid lethality was not conducive to experimental observation. The group injected with 1×10^8^ CFU/mL achieved progressive 100% mortality within 48 hours, meeting the requirement for controllable experimental progression (**Figure 8A** **).** Injections of 1×10^7^, 1×10^6^, and 1×10^5^ CFU/mL induced mortality with decreasing incidence rates of 26.67%, 13.33%, and 10%, respectively, with significant delays. Balancing lethal efficiency and the observation window, 1×10^8^ CFU/mL was selected as the standard infection concentration. In the experimental system, the survival rate of larvae in the PBS-treated group remained at 100%, with no abnormal pigmentation observed on the larval surface, confirming the non-toxicity of the buffer. To ensure experimental consistency, host bacteria were washed three times with PBS and resuspended, while phages were diluted using the same buffer to eliminate potential interference from residual culture medium.

This study evaluated the protective effects of phage vB_SmaS_QH16 against *G. mellonella* infection using therapeutic intervention and prophylactic protection models. In the therapeutic model, injecting phages at different multiplicities of infection (MOI, 0.1–100) following host bacterial infection significantly improved larval survival rates (*P* < 0.001). During the 72-hour observation period, survival rates exhibited a non-monotonic dose-dependent relationship. The highest survival rate (80.00%) was achieved at an MOI of 10, followed by 100 (70.00%) and 1 (66.67%). The survival rate at MOI = 0.1 was significantly reduced to 40.00%, contrasting sharply with the PBS control group, which exhibited no survival (**Figure 8B**). In the prophylactic intervention model, pre-injection of phages prior to bacterial challenge demonstrated superior protective effects. Survival rates at MOI = 10 remained at 80.00%, while the rates at MOI = 100, 1, and 0.1 were 76.67%, 73.33%, and 56.67%, respectively. Notably, MOI = 10 achieved the highest protective efficiency in both models, indicating that this MOI may represent an optimal intervention threshold *in vivo* (**Figure 8C**). Safety validation further confirmed that larvae injected with phage alone (without bacterial challenge) exhibited 100% survival and no observable abnormal phenotypes, indicating no toxicity of the phage to the host. Throughout the experiment, PBS was used as the buffer system, and the 100% survival rate of the PBS control group further validated the reliability of the experimental setup.

### 3.10 The therapeutic effects of vB_SmaS_QH16 in infected mice

**Figure 9:**
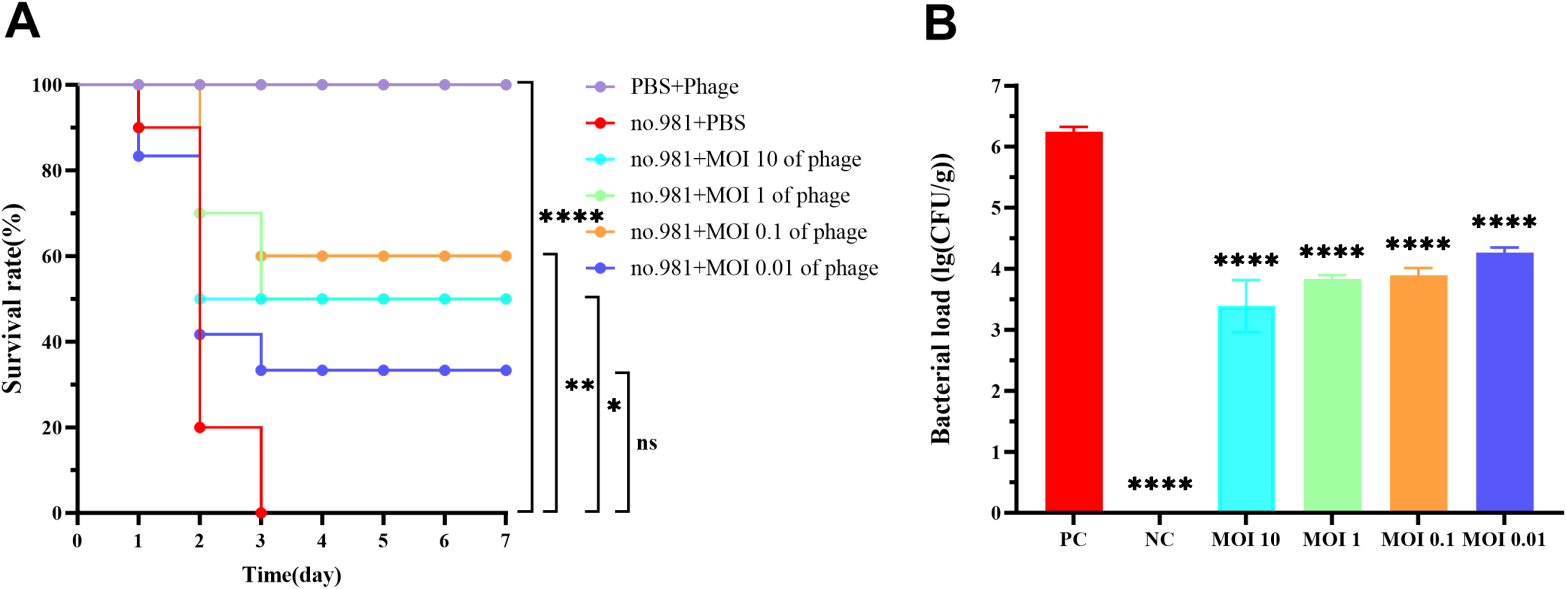
*In Vivo* bactericidal effects of vB_SmaS_QH16 in mice. **(A)** Survival curves of infected mice treated with phage vB_SmaS_QH16; **(B)** Bacterial load in the lungs of infected mice treated with phage vB_SmaS_QH16 at different MOIs. Symbols are defined as follows: ns, not significant; *P < 0.05; **P < 0.01; ****P < 0.0001.

Survival analysis revealed that all mice in the positive control group died within 3 days post-infection, whereas those in the negative control group survived throughout the experiment. In infected mice treated with varying concentrations of phages, mortality occurred during the early phase (days 1–2), but survival rates stabilized from day 3 onward. Compared with the positive control group, the phage-treated groups (MOI 10, 1, 0.1, and 0.01) demonstrated a significant enhancement in 7-day survival rates, with improvements of 50% (*P* < 0.05), 50% (*P* < 0.05), 60% (*P* < 0.01), and 20% (*P* > 0.05), respectively (**Figure 9A**). Bacterial load quantification demonstrated that 30 hours post - phage treatment, the pulmonary burden of *S. maltophilia* no. 981 in the positive control group reached 10^6^ CFU/g. In contrast, mice treated with higher phage titers (MOI = 10, 1, and 0.1) showed a marked reduction in bacterial loads to approximately 10³ CFU/g (*P* < 0.0001). Even at the lowest phage concentration (MOI = 0.01), the bacterial load was significantly reduced to 10^4^ CFU/g (*P* < 0.0001) (**Figure 9B**).

**Figure 10:**
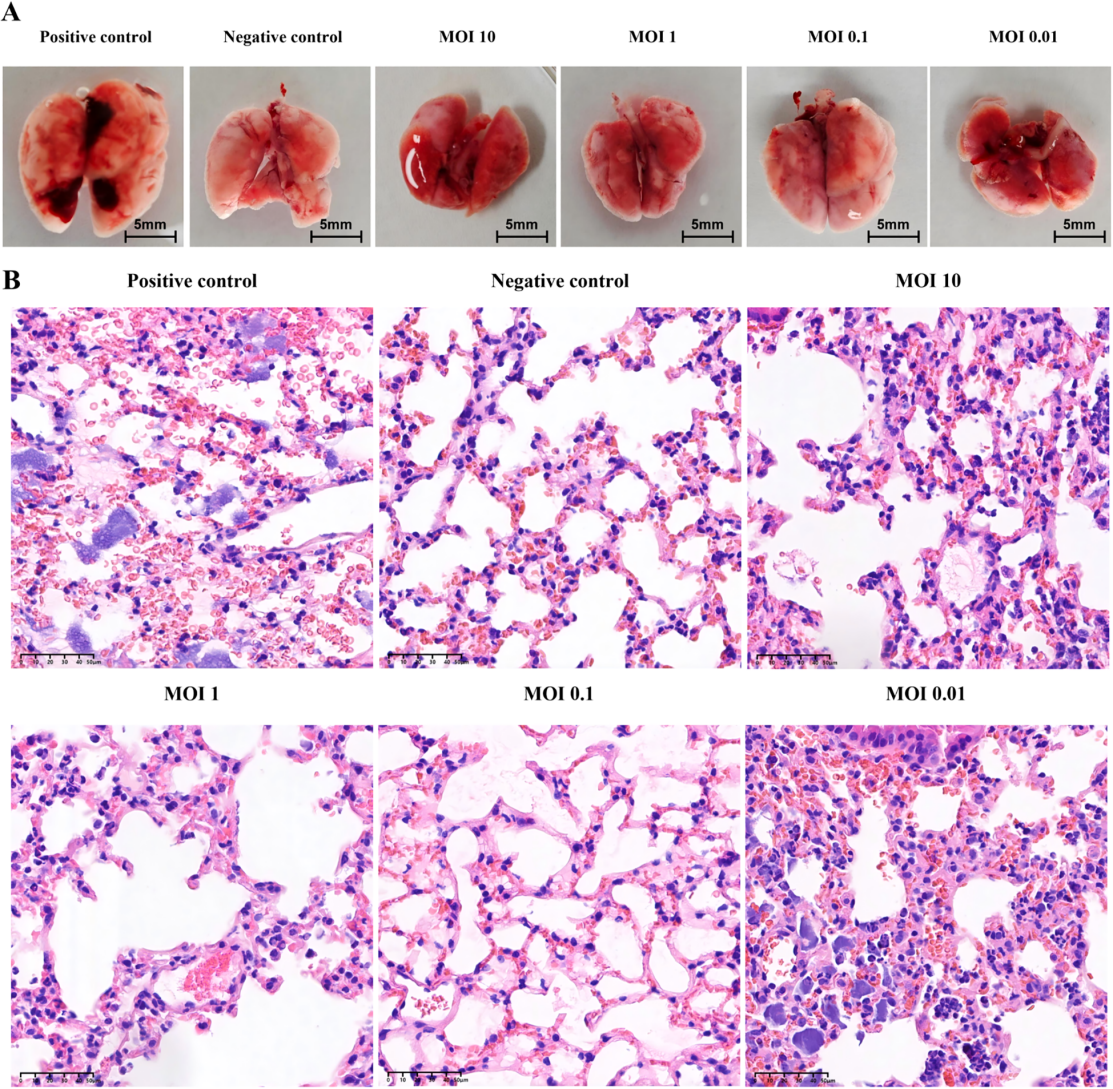
Pathological analysis of mouse lung in animal model. **(A)** Gross pathological features of mouse lung under different MOI and treatment conditions. **(B)** Analysis of pathological sections of mouse lung under different MOI and treatment conditions (H&E staining).

Gross examination of murine lung tissues revealed normal pulmonary morphology in the negative control group. In contrast, the positive control group exhibited marked pulmonary swelling with multiple hemorrhagic foci. All phage-treated groups (MOI=10, 1, 0.1, 0.01) showed varying degrees of congestion and edema, with only minor hemorrhagic spots observed in the MOI=0.01 group. Notably, the overall pathological severity in all treatment groups was substantially milder than in the positive controls (**Figure 10A**). Histopathological examination further confirmed that the positive control group’s lung parenchyma had multiple scattered bacterial colonies along with lymphocytic and neutrophilic infiltration. In contrast, higher-titer phage treatment (MOI = 10, 1, 0.1) significantly alleviated these pulmonary pathological changes (**Figure 10B**).

## 4 Discussion

In recent years, lytic phages have garnered renewed attention as promising natural alternatives to antibiotics, particularly for the treatment of severe drug-resistant bacterial infections (Alqahtani, 2023). Isolating phages with a strict lytic cycle from nature and comprehensively evaluating their biological characteristics, physicochemical stability, antibacterial efficacy, and biosafety are critical steps in transitioning phage research from basic science to clinical application (Abedon et al., 2017; Venturini et al., 2022). In this study, we successfully isolated a lytic phage from hospital sewage, designated vB_SmaS_QH16 (GeneBank accession number: PP921744). In double-layer plate assays, this phage formed clear, transparent plaques with diameters ranging from 1 to 2 mm, directly confirming its lytic nature. Additionally, in-depth genomic analysis revealed no genes related to lysogeny. Furthermore, predictions of its lifestyle using a specialized phage AI tool classified it as “Virulent.” Collectively, these findings confirm that vB_SmaS_QH16 exhibits a strict lytic lifestyle, which is of practical importance, as non-lysogenic phages are typically preferred for therapeutic applications due to their inability integrate into the host bacterial genome, thereby avoiding potential safety risks (Venturini et al., 2022). Additionally, no virulence or antibiotic resistance genes were predicted in the genome of vB_SmaS_QH16, molecularly confirming its biosafety.

Phage vB_SmaS_QH16 demonstrated significant lytic activity in lytic assays targeting 73 strains of *S. maltophilia*, successfully lysing 35 (47.95%) of the tested strains. Further analysis revealed that the lysed strains covered 13 distinct STs, including ST4, ST8, ST115, ST138, ST185, ST208, ST212, ST223, ST365, ST517, ST518, ST644, and ST1139. Notably, most of these STs of *S. maltophilia* have been reported in clinical patients or hospital settings (Kaiser et al., 2009; Li et al., 2023), indicating that this phage holds significant promise for the prevention and control of hospital-acquired infections. Moreover, *S. maltophilia* no.981 was isolated from a patient’s bloodstream infection sample. Studies indicate that *S. maltophilia* - related bloodstream infections can have a mortality rate as high as 69% (Brooke, 2012). Thus, selecting *S. maltophilia* no.981 as the host strain is of great clinical significance for phage screening and evaluating phage antibacterial effects.

The optimal multiplicity of infection for phage vB_SmaS_QH16 was 0.01, indicating a high lytic activity at low infection ratios, and implying that a low initial dose could achieve therapeutic effects (Bai et al., 2022). Its latent period was approximately 40 minutes, with a lytic phase of 60 minutes. This short life cycle enables this phage to rapidly lyse host bacterial populations and effectively control infections. Its average burst size is 37.69 PFU/cell, comparable to those of vB_ SmaS_QH3 (41.67 PFU/cell) (Cheng et al., 2025) and A1432 (43.2 PFU/cell) (Li et al., 2024), and slightly higher than that of StM171 (12 PFU/cell) (Jdeed et al., 2023).

To ensure the efficacy of phage vB_SmaS_QH16 in clinical applications and its storage efficiency, we evaluated its stability under different physicochemical conditions. Our results showed that the phage remained stable between 4°C and 50°C, although its titer gradually decreased with increasing temperature. Even after 1 hour at 80°C, it did not fully inactivate, demonstrating good thermal tolerance that meets the clinical and ambient storage requirements. Its biological activity remained stable after 1 hour at pH 3.0 to 11.0. However, at extreme pH levels (1, 13, and 14), its activity significantly decreased or fully inactivated. Given that the pH values of human blood and gastric juice are 7.35–7.45 and 1–2, respectively (Efentakis and Dressman, 1998), the phage’s activity is significantly affected in gastric juice. Thus, clinical use should avoid gastric administration, or may require protection of the phage with alginate and chitosan to prevent gastric inactivation. UV irradiation for 10 minutes significantly reduced activity, with near-complete inactivation after 30 minutes, indicating the need to avoid UV exposure during handling and transport. The phage also showed good resistance to chloroform, which can remove bacterial contaminants during preparation, ensuring phage purity (Mäntynen et al., 2019). Overall, the phage’s favorable physicochemical stability facilitates its activity in diverse environments, enhancing clinical application and storage.

The genome of phage vB_SmaS_QH16 is a 43,500 bp dsDNA containing 64 ORFs, with a gene density of 95.93%, enabling it to encode sufficient functional genes in a compact genome, thereby reducing replication costs (Dion et al., 2020). The efficient expression of phage genes allows rapid synthesis and function of lysis-related proteins, which crucial for the phage life cycle, as it directly impacts reproductive efficiency and spread. In most phages of Gram-negative hosts, the main lytic players are holins, endolysins, and spanins (Cahill and Young, 2019). The lysis module of vB_SmaS_QH16 comprises an endolysin (ORF39), a holin/antiholin class IV (ORF40), a holin/antiholin class I (ORF41), an i-spanin (ORF42), and an o-spanin (ORF43), forming a gene cluster that works in concert to ensure precise host lysis. In the late stage of infection, holin expression is triggered, forming nonspecific “pores” in the cell membrane. Antilysin inhibits premature pore formation, ensuring lysis occurs at the optimal time, under the control of the holin/antiholin system (ORF40 and ORF41). Pore formation activates the endolysin, allowing it to escape and destroy the peptidoglycan in the cell wall. The endolysin transcribed from ORF39 shares 79.2% homology with that of Stenotrophomonas phage SM171 (MZ611865). Additionally, spanins, which are complexes of an outer membrane lipoprotein (o-spanin) and an inner membrane protein (i-spanin), are activated by peptidoglycan degradation, leading to outer membrane disruption and release of progeny phages (Young, 2014).

Comparative genomics and phylogenetic analyses are crucial for phage classification (Lefkowitz et al., 2017). In the present study, phage vB_SmaS_QH16 showed homology only with four phages in the NCBI database: StM171 (MZ611865.1), HXX_Dennis (ON711490.1), and Suso (MZ326866.1). Its maximum similarity to phage StM171 was 79.37%, which is below the ICTV’s 95% ANI threshold for defining a new species (Lefkowitz et al., 2017), suggesting vB_SmaS_QH16 is a novel phage species. VIRIDIC-based ANI analysis further showed only 59.5% ANI between vB_SmaS_QH16 and its closest relative, HXX_Dennis, which is far below the 70% ANI threshold for genus demarcation (ICTV 2023), indicating vB_SmaS_QH16 should be classified as a new species in a new genus. Phylogenetic analysis using VICTOR further showed that vB_SmaS_QH16 forms a distinct clade. The conserved phage proteins, terminase large subunit and major capsid protein (Kang et al., 2022), were used for phylogenetic analysis, revealing that vB_SmaS_QH16 clusters with class Caudoviricetes phages. This aligns with its morphological features observed under electron microscopy, supporting its classification as a novel phage within class Caudoviricetes.

We further found that phage vB_SmaS_QH16 inhibited the growth of *S. maltophilia* no.981 *in vitro* broth culture in a MOI-dependent manner, with 24-hour inhibition rates 59.61%, 67.77%, 68.77%, 76.63%, 81.12%, 83.56%, and 80.66% at MOIs of 100, 10, 1, 0.1, 0.01, 0.001, and 0.0001, respectively, with better inhibition at lower phage concentrations (MOI 0.01–0.0001). Growth curves showed that high phage concentrations (MOI 100 –1) led to increased OD_600_ values after approximately 10 hours, indicating the emergence and proliferation of phage-resistant mutants. In contrast, inhibition curves for low phage concentrations (MOI 0.1–0.0001) showed initial increases followed by decreases, reflecting a “phage-bacteria arms race”. These results indicate that while phages effectively inhibit bacterial growth, resistant strains eventually emerge, and their appearance is earlier in high phage concentration groups. This aligns with prior observations of phages vB_Kpn_ZC2 (Fayez et al., 2023) and BUCT700 (Li et al., 2023). This phenomenon indicates a complex dynamic balance between phage concentration and bacterial resistance evolution. Future research should investigate the kinetics of bacterial resistance mutations at different phage concentrations to optimize phage therapy strategies, to provide a theoretical basis and new directions for clinical phage applications and combination therapies.

Biofilms are implicated in 65%–80% of bacterial infections (Gupta et al., 2015; Visnapuu et al., 2022), and bacteria within biofilms are 10- to 1000-fold more tolerant to conventional antibiotics than planktonic bacteria, making biofilm formation a key factor driving antibiotic resistance. Phages have shown significant potential in biofilm inhibition and removal (Chegini et al., 2020; Visnapuu et al., 2022; Mayorga-Ramos et al., 2024). In the present study, phage vB_SmaS_QH16 prevented *S. maltophilia* biofilm formation and disrupted mature biofilms. Low phage concentrations were more effective for prevention, while high concentrations were better for removal. This contrasts with most phages, where higher concentrations typically offer better biofilm inhibition, sometimes with no effect at low concentrations (Yang et al., 2022). This difference may stem from the rapid phage resistance developed by *S. maltophilia* no.981 against vB_SmaS_QH16. Fluorescence microscopy showed more bacteria in high MOI (100–1) groups during biofilm formation experiments than in low-concentration phage groups, consistent with *in vitro* inhibition results, indicating high phage concentrations more readily induce resistant strains. Overall, phage vB_SmaS_QH16 effectively inhibited bacterial growth, prevented biofilm formation, and removed existing biofilms *in vitro*, highlighting its potential against biofilm-associated infections. Future research should explore phage-biofilm interaction mechanisms and optimize infection concentrations and phage pharmacokinetics to maximize its antibacterial efficacy.

This study employed *G. mellonella* and mouse models to validate the protective effect of phage vB_SmaS_QH16 against *S. maltophilia* infection and found that the optimal dosing strategy is host - specific. In the *G. mellonella* model, the highest survival rate of 80% (for both therapeutic and prophylactic treatments) was achieved at MOI 10, while the protective effect decreased at MOI 100. This might be due to the release of toxic bacterial components (e.g., endotoxins) from phage - lysed bacteria, which may have harmed the *G. mellonella* larvae (Pu et al., 2022). In the mouse model, phage treatment at MOI ≥ 0.01 significantly increased the survival rate (20% - 60%) and reduced the pulmonary bacterial burden by approximately 1000 - fold to about 10³ CFU/g in high - MOI groups. Moreover, histopathological evaluation revealed significantly reduced pathological damage at MOI ≥ 0.1. Conversely, only a 20% increase in survival rate was observed at MOI 0.01, indicating that underdosing may compromise treatment efficacy. Both models showed potential adverse effects of ultra - high MOI. In the *G. mellonella model*, the survival rate of the MOI 100 group dropped to 70%, implying excessive phages might harm the host via resource competition or metabolic stress. This is highly relevant for designing clinical doses for immunocompromised patients like those undergoing chemotherapy, emphasizing the need to balance bactericidal efficiency with host tolerance. Additionally, both models confirmed the phage’s safety, with 100% survival in phage - only groups, which matches literature reports (Wang et al., 2024). This safety data is crucial for clinical translation, as safety is a prerequisite for clinical use. In summary, phage vB_SmaS_QH16 is a promising safe and effective alternative for treating *S. maltophilia* infections but requires strict dose control due to the close dose - efficacy relationship. The differing phage mechanisms across host models highlight the importance of diverse animal model systems, and future cross - species studies are necessary to determine the human equivalent dose.

## 5 Conclusions

In this study, we isolated a novel lytic phage, designated as vB_SmaS_QH16, from hospital sewage. Functional characterization revealed that this phage can lyse multiple *S. maltophilia* sequence types, showing a broad host range and a safe genome profile. The phage is stable under diverse physicochemical conditions, efficiently lysed bacteria *in vitro*, and effectively inhibited and removed biofilms. It also demonstrated antimicrobial efficacy *in vivo*, highlighting its promising application potential. However, in clinical applications, gastric administration and UV exposure should be avoided. Additionally, appropriate phage doses must be selected to balance bacteriolytic effects and resistance development. Future research could explore phage synergy with other antimicrobials to enhance therapeutic outcomes and prevent resistance. Furthermore, in-depth studies on phage mechanisms against biofilm-associated infections are required to provide comprehensive theoretical support for clinical use.

## Data Availability

The original contributions presented in the study are publicly available. This data can be found here: https://www.ncbi.nlm.nih.gov/nuccore/PP921744.
