# Supplementary material for: Characterization and antimicrobial activity of a novel lytic phage vB_SmaS_QH16 against Stenotrophomonas maltophilia: in vitro, in vivo, and biofilm studies
Source: Front Cell Infect Microbiol. 2025 Jul 10;15:1610857. doi: 10.3389/fcimb.2025.1610857 (PMC12286969; doi:10.3389/fcimb.2025.1610857)
Supplement: Supplementary file 1 [file DataSheet1.docx]

**
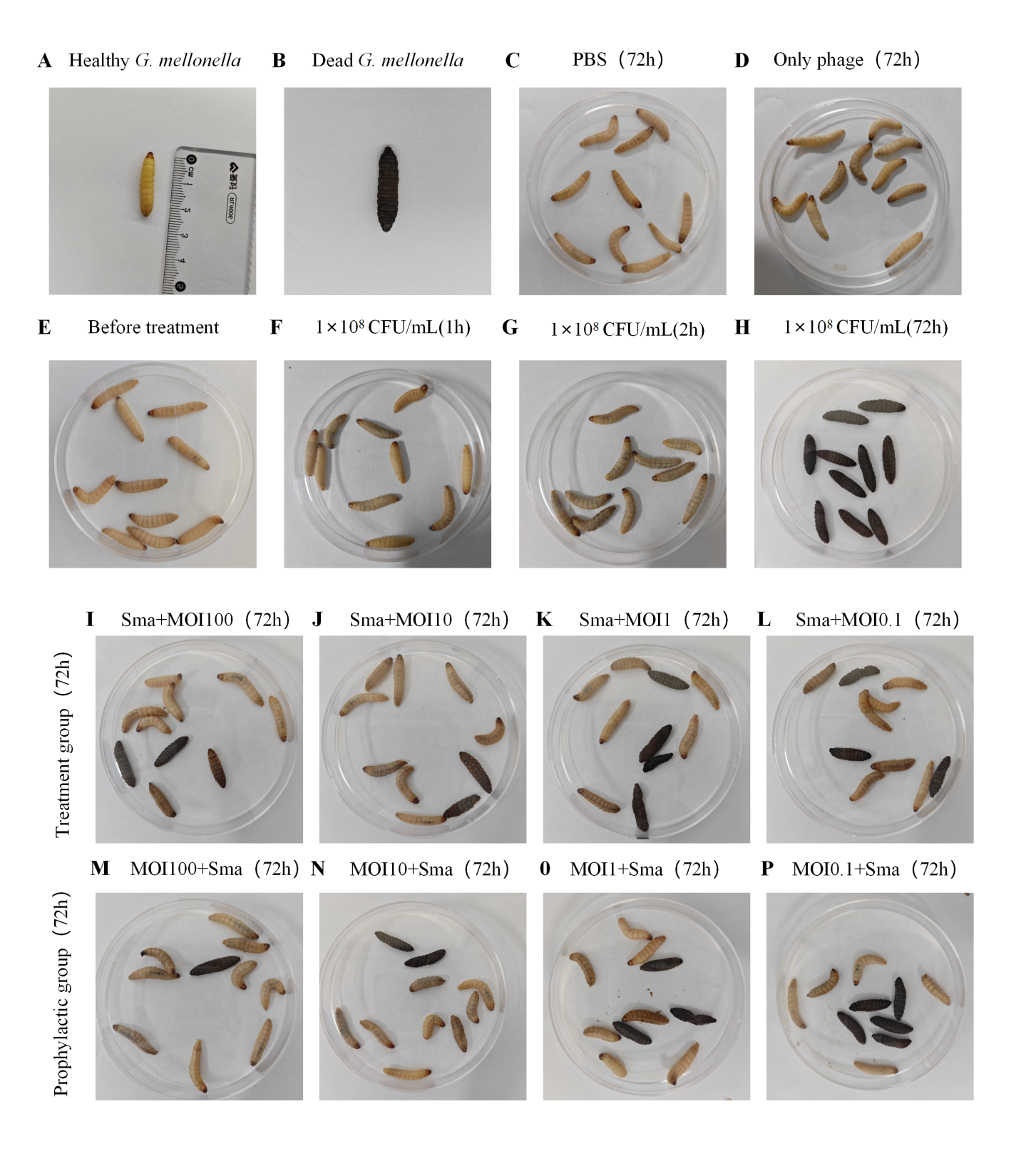
**

**Supplementary Figure 1.**  Morphological changes of wax moth larvae; **(A)** Healthy *G. mellonella* larvae: milky white in color, smooth surface, no spots; **(B)** Larvae that died from infection: black in color, non-responsive to touch; **(C-D)** After injection of 10 μL PBS or phage vB_SmaS_QH16, no significant abnormalities were observed in the larvae after 72 hours; **(E-H)** Infected larvae: Larvae were in a normal state before bacterial infection **(E)**, 1 hour **(F)**, 2 hours **(G)**, and 72 hours **(H)** post-infection, larvae gradually showed melanization and eventually died; **(I-L)** Phage treatment groups at different MOI (multiplicity of infection); **(M-P)** Phage prophylaxis groups at different MOI.
